# Effect of yield locus exponent on draw-in prediction during deep drawing of commercially pure titanium

**DOI:** 10.1038/s41598-025-23156-y

**Published:** 2025-11-11

**Authors:** Lukas Gassler, Andreas Hirsch, Mohamadreza Afrasiabi

**Affiliations:** 1 Advanced Manufacturing Lab, ETH Zurich, 8005 Zurich, Switzerland; 2 Computational Manufacturing Group, Inspire AG, 8005 Zurich, Switzerland

**Keywords:** Yield locus, Parameter identification, Sheet metal forming, Titanium, Engineering, Materials science

## Abstract

The Yld2000-2D yield locus is widely employed to model anisotropic plasticity in sheet metal forming. Its exponent, *a*, critically influences the shape of the yield surface and is typically assigned fixed values based on crystal structure–commonly $a=6$ for body-centered cubic (BCC) and $a=8$ for face-centered cubic (FCC) materials. However, no universally accepted value exists for hexagonal close-packed (HCP) metals such as commercially pure titanium, which exhibit pronounced anisotropy and complex hardening behavior. This study explores the influence of the value of *a* on accurately modeling the forming response of commercially pure titanium sheets. To examine the impact of the exponent value a comparison between finite element (FE) simulations and experimental draw-in profiles obtained from cup-drawing tests is performed. To account for evolving anisotropy during plastic deformation, the Yld2000-2D yield locus is further augmented with strain-dependent coefficients, while the loading asymmetry commonly present in HCP metals is neglected. The results show a clear preference for larger exponent values with $a>9$ giving the best results. This highlights the necessity of material-specific calibration for HCP alloys and provide actionable insights for improving the predictive fidelity of titanium sheet forming simulations.

## Introduction

Accurate modeling of sheet metal forming processes is essential for the reliable design and optimization of lightweight structures, especially in industries such as aerospace and automotive engineering. Commercially pure titanium, with its excellent strength-to-weight ratio and corrosion resistance, has become a material of great interest. However, titanium’s pronounced anisotropy, stemming from its hexagonal close-packed (HCP) crystal structure, poses significant challenges for predictive simulations. Capturing the complex deformation behavior of titanium sheets demands advanced constitutive models and careful calibration of key material parameters. Among these, the correct determination of the yield locus shape is critical for achieving accurate and robust forming predictions.

In modeling sheet metal forming processes, the yield locus plays a crucial role in capturing the material’s anisotropic behavior. Various formulations of yield loci exist, ranging in complexity and accuracy – see^1^ for more insights. While more sophisticated models can better describe material behavior, they typically require additional experimental data for parameter calibration and validation. One parameter that is often left uncalibrated is the exponent of the yield locus formulation, usually denoted as *a* in the literature. Logan and Hosford^2^ proposed fixed exponent values of $a=6$ for body-centered cubic (BCC) and $a=8$ for face-centered cubic (FCC) materials. These recommendations have been widely adopted in both academia and industry, even though subsequent studies, such as the works of Pilthammar et al.^3^ and Barth et al.^4^, have shown that optimizing *a* can improve the agreement between predicted and experimental strain distributions.

For materials with HCP crystal structures, such as titanium, no analogous recommendation exists. Titanium alloys and commercially pure titanium exhibit significant anisotropy, which arises from the crystallographic texture developed during processing. Despite this, many constitutive models for titanium forming processes assume a fixed $a=8$, a value originally proposed for FCC materials. Previous investigations into the mechanical behavior of HCP materials, such as those by Davies et al.^5^ and Pham et al.^6^, indicate that the anisotropy in titanium cannot be accurately captured using yield locus exponents derived from BCC or FCC analogies. Furthermore, titanium displays anisotropic hardening–or more precisely, isotropic differential (or distortional) hardening–during plastic deformation, a phenomenon discussed in detail by Pham et al.^6^ and Kim et al.^7^. Similar effects have been successfully modeled for other materials, such as DC05 steel^8^ and 5754-O aluminum^9^, emphasizing the need for careful modeling strategies in the case of titanium.

To address these complexities, advanced asymmetric yield criteria, such as the Barlat-Cazacu model^10^ and the Yoon asymmetric yield function^11^, have been developed. These models are capable of capturing tension-compression asymmetry and more complex anisotropic behaviors but require an extensive set of material tests, including additional shear and compression experiments, for full calibration. Given the practical limitations in acquiring such comprehensive experimental data, particularly for thinner materials, there is a strong motivation to pursue approaches that balance accuracy with experimental feasibility. In this context, the Yld2000-2D yield function stands out, as it provides a good compromise: it can accurately describe anisotropy based on limited standard tests (three tensile directions and a bulge test) while still being flexible enough to capture strain-dependent material behavior when extended with anisotropic hardening formulations.

Badr et al.^12^ conducted an initial investigation into the optimal exponent of the Yld2000-2D yield function for Ti-6Al-4V sheet material. They evaluated the values 6, 8, and 12 based on their ability to predict initial yielding at the plane-strain point, ultimately concluding that an exponent of 12 provided the best agreement with experimental data. Although the material was predominantly composed of the $\alpha$-phase (characteristic of an HCP structure), the presence of some $\beta$-phase (BCC structure) was also reported^13^, potentially influencing the optimal exponent. Furthermore, the study focused solely on specific points of the initial yield surface, without assessing broader deformation paths or strain evolution during forming.

Building upon this motivation, the present work evaluates the predictive capabilities of the Yld2000-2D yield locus with plastic strain-dependent parameters, as presented in^9^, by systematically tuning the exponent *a* for commercially pure titanium sheets. The effect of the exponent value is examined using experimental data from a series of cup-drawing tests. By comparing the predicted and measured draw-in profiles, the suitability of different *a*-values for accurately modeling the anisotropic behavior of HCP materials is determined. The findings provide practical recommendations for enhancing the reliability of titanium forming simulations while maintaining a manageable experimental effort.

The remainder of this manuscript is structured as follows. Section "Methods" introduces the methodology, including the formulation of the Yld2000-2D yield locus along its calibrated parameters, the experimental setup for the cup-drawing tests, the measurement procedures, and the FE simulation framework used for the inverse calibration. Section "Results and discussion" presents the results, comparing experimental observations with simulation outcomes and analyzing the influence of different exponent values on the predicted material response. It also contains a succinct discussion that contextualizes the results and reflects on the broader implications of our study. Finally, Section "Conclusion" concludes the study by summarizing the key findings and proposing directions for future work.

## Methods

### Yld2000-2D yield locus

The Yld2000-2D yield locus, introduced by Barlat et al.^14^, is a non-quadratic yield criterion expressed mathematically as:

1 $$\begin{aligned}&\Phi = \Phi ' + \Phi '' = 2 \bar{\sigma }^a \end{aligned}$$

2 $$\begin{aligned}&\Phi '=|X_1' - X_2'|^a \end{aligned}$$

3 $$\begin{aligned}&\Phi ''=|2 X_2'' + X_1''|^a + |2 X_1'' + X_2''|^a \end{aligned}$$

where $\bar{\sigma }$ is the equivalent stress, $X_i'$ and $X_i''$ are the principal values of two linear transformations of the plane stress tensor:

4 $$\begin{aligned} X' = \begin{bmatrix} X_{11}' & X_{12}' \\ X_{12}' & X_{22}' \\ \end{bmatrix} \end{aligned}$$

with its entries defined as follows:

5 $$\begin{aligned} \begin{bmatrix} X_{11}' \\ X_{22}' \\ X_{12}' \end{bmatrix} = \begin{bmatrix} L_{11}' & L_{12}' & 0 \\ L_{21}' & L_{22}' & 0 \\ 0 & 0 & L_{66}' \end{bmatrix} \begin{bmatrix} S_{xx} \\ S_{yy} \\ S_{xy} \end{bmatrix} \end{aligned}$$

where $S_{ij}$ are the components of the deviatoric stress tensor. The definition of $X''$ is defined analogously. The entries of the two operators $L'$ and $L''$ are further obtained as:

6 $$\begin{aligned} & \begin{bmatrix} L_{11}' \\ L_{12}' \\ L_{21}' \\ L_{22}' \\ L_{66}' \end{bmatrix} = \begin{bmatrix} \frac{2}{3} & 0 & 0 \\ -\frac{1}{3} & 0 & 0 \\ 0 & -\frac{1}{3} & 0 \\ 0 & \frac{2}{3} & 0 \\ 0 & 0 & 1 \end{bmatrix} \begin{bmatrix} \alpha _1 \\ \alpha _2 \\ \alpha _7 \end{bmatrix} \end{aligned}$$

7 $$\begin{aligned} & \begin{bmatrix} L_{11}'' \\ L_{12}'' \\ L_{21}'' \\ L_{22}'' \\ L_{66}'' \end{bmatrix} = \frac{1}{9} \begin{bmatrix} -2 & 2 & 8 & -2 & 0 \\ 1 & -4 & -4 & 4 & 0 \\ 4 & -4 & -4 & 1 & 0 \\ -2 & 8 & 2 & -2 & 0 \\ 0 & 0 & 0 & 0 & 9 \end{bmatrix} \begin{bmatrix} \alpha _3 \\ \alpha _4 \\ \alpha _5 \\ \alpha _6 \\ \alpha _8 \end{bmatrix} \end{aligned}$$

These transformations are governed by eight model parameters ($\alpha _1,\ \alpha _2,\ ...,\ \alpha _8$) and the yield locus exponent *a*. To calibrate the eight $\alpha$-values, eight material parameters are required. Following the standard procedure described in^14^, we employed the yield stresses and Lankford coefficients obtained from tensile tests conducted at $0^\circ$, $45^\circ$, and $90^\circ$ to the rolling direction, as well as data from a hydraulic bulge test. This provides eight material values, which yield a system of eight non-linear equations to be solved for the parameter set. In the present study, this system was solved using the MATLAB fsolve function^15^. Default convergence settings were applied, except for the termination tolerances, which were set to $10^{-18}$ for the function value and $10^{-12}$ for the change in the current point. These settings consistently provided robust and reproducible parameter values throughout the study. Finally, to ensure the correct calibration, simulations of the material experiments were performed to check whether the obtained material parameters could be replicated.

To determine the optimal value of the exponent *a*, the model parameters are calibrated for nine different values of *a*, ranging from a=4 to a=12. Larger exponent values were not included in the investigation due to the negligent influence on the yield locus model, this is visualized in Fig. 9 where the resulting yield locus with an exponent of a=16 is also included to show the diminishing changes of large exponent values on the yield locus shape. Additionally, due to using the associated flow rule, larger values for the yield locus exponent could potentially cause numerical instability when used for FE simulations, which is why this investigation restricts itself to maximum exponent value of a=12. The same set of experimental data is used consistently across all calibration runs. Specifically, the yield stresses and Lankford coefficients are evaluated at different effective plastic strains. The parameter identification procedure follows the methodology outlined in^14^.

To capture the material’s hardening behavior, a Hockett–Sherby hardening law^16^ is employed, which is defined as follows:

8 $$\begin{aligned} k_f = \sigma _{sat} - (\sigma _{sat} - \sigma _{yld}) e^{-m \varepsilon _{pl}^n} \end{aligned}$$

where $k_f$ is the yield stress, $\varepsilon _{pl}$ is the effective plastic strain and $\sigma _{sat}, \sigma _{yld}, m$, and *n* are the hardening function parameters.

### Material tests

To obtain the material data required for calibration, tensile specimens were prepared in accordance with DIN ISO 50125, shape H^17^, using wire-cutting. The tensile tests were conducted and evaluated following the procedures outlined in ISO 6892-1^18^ and ISO 10113^19^, ensuring standard-compliant assessment of mechanical properties.

In addition, a hydraulic bulge test with a die diameter of 100 mm was performed in accordance with ISO 16808^20^ to determine the biaxial flow stress ($\sigma _b$) and the plastic strain ratio under biaxial loading conditions ($r_b$).

### Cup-drawing experiment

The cup-drawing experiment is a well-established benchmark test in sheet metal forming, commonly employed to evaluate the planar anisotropy of sheet materials. In this study, the standardized earing test^21^ was adapted to halt the drawing process before full cup formation. This modification simplifies the quantitative evaluation of the draw-in profile by focusing on the partially drawn cup geometry, thereby enhancing the efficiency and accuracy of anisotropy characterization.

**Fig. 1:**
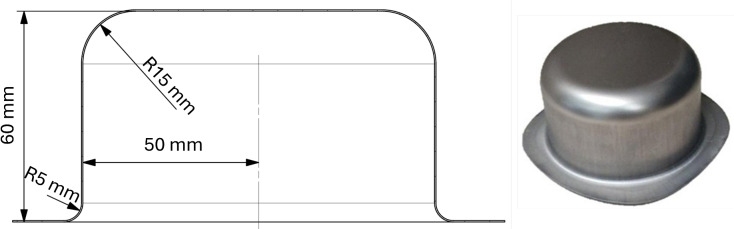
The schematic drawing and actual geometry of the titanium cup produced during this study.

**Fig. 2:**
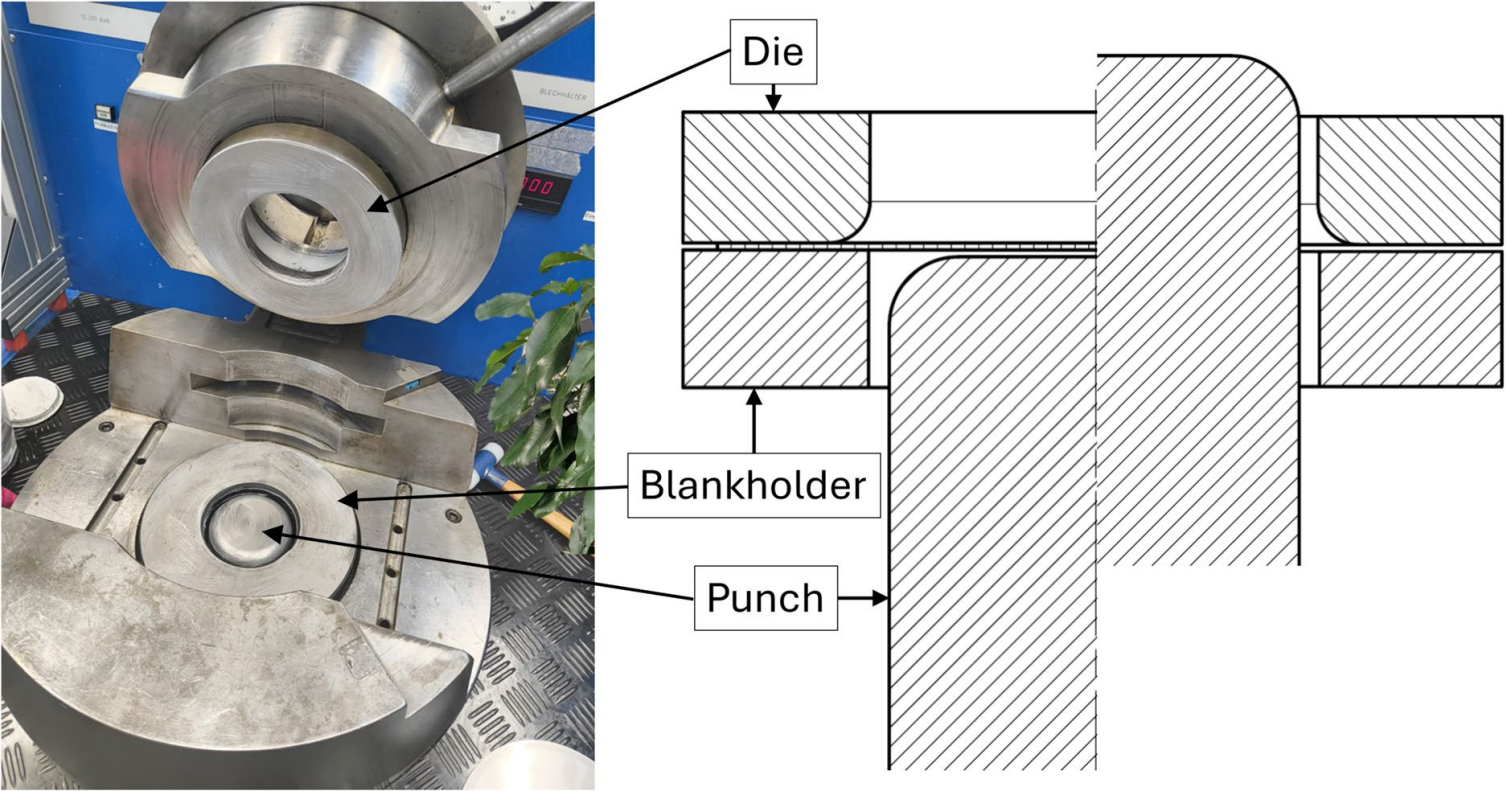
Sheet metal testing machine with installed cup drawing tools.

The geometry and dimensions of the manufactured cup are shown in Fig. 1. The blank used in the forming process was a circular sheet with a diameter of 190 mm and an initial thickness of 0.4 mm. To minimize friction, a Molykote EM-30 L lubricant was applied to all tooling surfaces. The tooling setup consisted of a die, a punch, and a blank holder, as illustrated in Fig. 2. A blank-holder force of 110 kN was applied to prevent wrinkle formation in the flange area.

### Measurement of draw-in profile

**Fig. 3:**
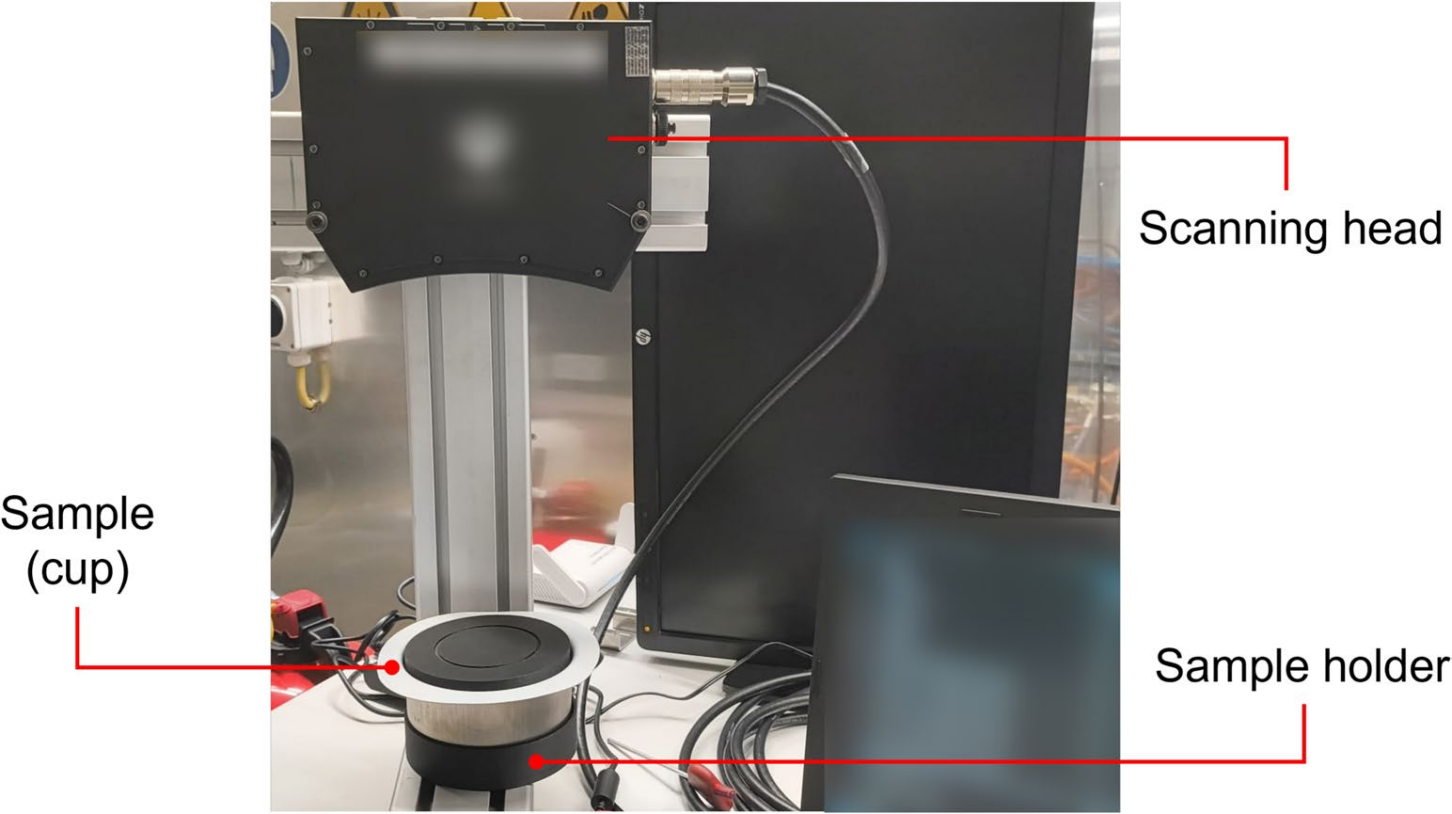
Measurement setup equipped with a Gocator optical sensor used to scan the surface of the final part after cup draw-in.

The geometric evaluation of the drawn cups was conducted using blue light strip scanning with a Gocator 3210 scanner from LMI Technologies. The parts were mounted on a custom bracket and aligned so that the rolling direction of the sheet coincided with the scanner’s *xy*-axis, ensuring repeatable measurements. To aid alignment and reduce reflective aliasing, an insert with an indented circle was manufactured, and a non-reflective paint was applied to the parts. The setup is depicted in Fig. 3.

The scanning parameters are as follows:

- Spatial resolution: 50 $\mu$m (the finest resolution supported by the scanner).
- Exposure times: 250, 2500, and 10000 $\mu$s. These scans were overlaid to enhance measurement accuracy.

**Fig. 4:**
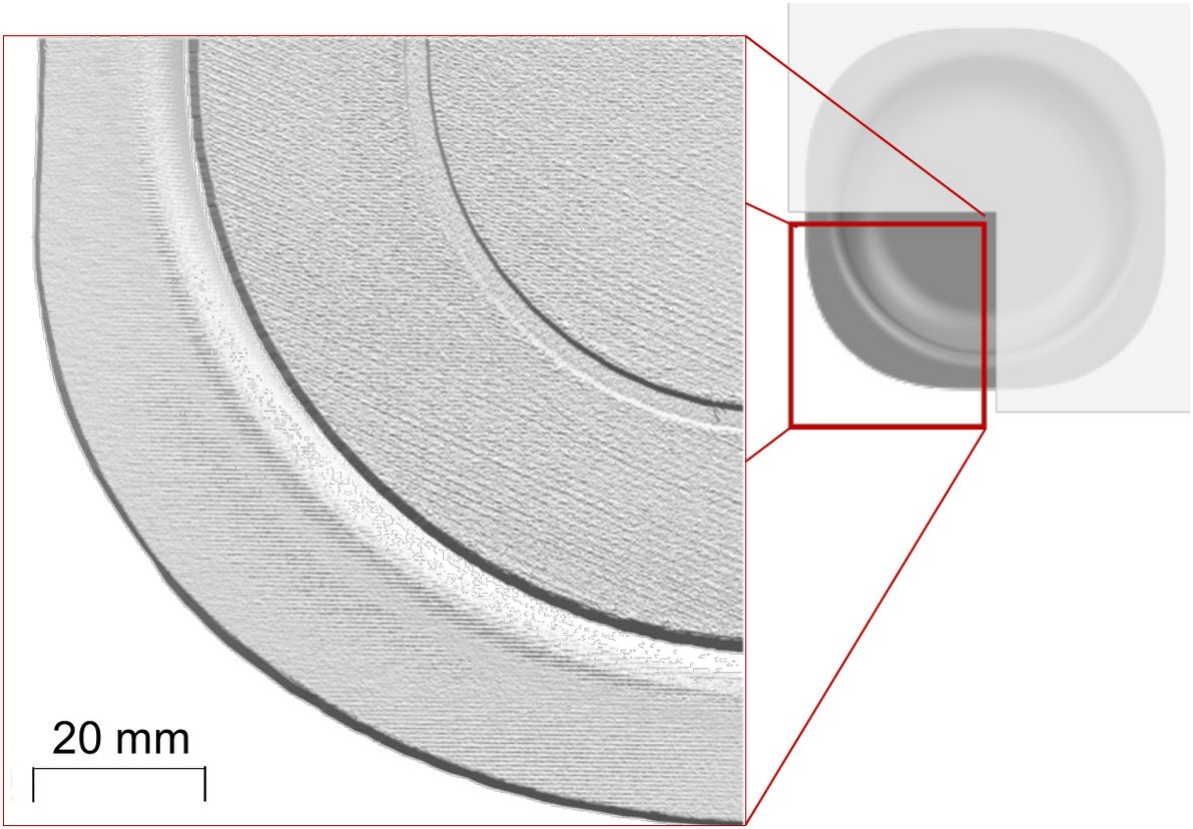
Top view of a magnified quadrant of the scanned part resulting from the optical sensor acquisition.

The resulting point cloud data was converted into an STL file for further analysis. Fig. 4 shows a top view of a quadrant of the scanned part. The indented circle with a radius of 30 mm to aid alignment, marked with a red arrow, serves as a reference geometry for subsequent analysis. Surface waviness artifacts are also visible in the scan but are not relevant to this study.

From the scanned geometry, the cup’s outline was extracted and compared to the corresponding outline obtained from the simulation. To assess the model’s accuracy in predicting material flow during the drawing process, the radial error of the predicted outline was calculated within the section [$0^\circ$, $90^\circ$], assuming geometric symmetry. The total error was quantified as the mean square error (MSE) of all measuring points along the outline, defined as:

9 $$\begin{aligned} e_{tot} = \frac{1}{N_p} \sum \left( r_{exp} - r_{sim}\right) ^2 \end{aligned}$$

where $N_p$ represents the total number of measurement points.

### Simulation of cup-drawing experiment

To generate predictive results using the various material models, an explicit FE-based simulation framework was developed in LS-Dyna. The tool surfaces were modeled as rigid bodies, while the titanium sheet was represented using deformable shell elements based on the formulation by Belytschko et al.^22^. For friction, the coulomb friction model with a friction coefficient of $\mu =0.08$ was used. Although more complex friction models exist (see for example^23,24^), but usually require additional calibration experiments and sometimes also the writing of a user-subroutine depending on the FE-solver used. As a result, the coulomb friction model is still widely applied in industry. Additionally, potential errors arising from the use of this simplified friction model should be consistent among the tested yield locus exponents and should therefore not affect their relative performance. To reduce computational cost and optimize efficiency, mass scaling was applied to the model. To ensure the applied mass scaling did not create any unwanted dynamical effects, the timestep was set, so that the kinetic energy of the model at no point exceeded 1% of the internal energy. The remaining mechanical properties were assumed from the datasheet provided by the supplier as well as measured during the tensile tests and are summarized in Table 1. The mesh size was set to a global size of 0.6 mm to ensure that the cup radii are captured in high enough detail. The element formulation used was LS-Dyna’s shell element with seven through-thickness integration points. This enables the model to properly capture the through thickness stress distribution due to bending deformation.

**Table 1:** Properties of Grade 1 pure titanium sheet used in this study.

Parameter	Value	Unit	Ref.
Young’s modulus	100	GPa	Measured
Poisson’s ratio	0.33	–	Datasheet
Density	4510	kg/m^3^	Datasheet

## Results and discussion

**Fig. 5:**
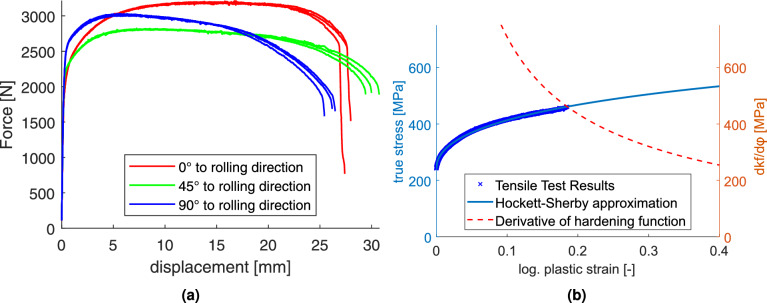
(**a**) Experimentally measured force-displacement curves for all tensile directions. (**b**) Yield stress evolution in the rolling direction with the corresponding Hockett-Sherby fit and its derivative.

The force-displacement responses obtained from the uniaxial tensile tests are illustrated in Fig. 5a. The results clearly indicate anisotropic hardening behavior, with significant directional dependence observed across the tested orientations. The calibrated hardening function for the $0^\circ$ direction is showcased Fig. 5b.

### Material data

**Fig. 6:**
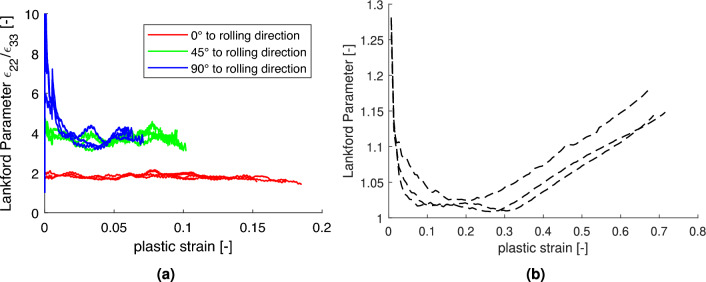
(**a**) Evolution of the Lankford coefficients for the three uniaxial tensile directions. (**b**) Evolution of the Lankford coefficients during the equi-biaxial bulge test.

Figure 6 presents the Lankford coefficients (*r*-values) determined from the tensile and bulge tests. As shown in Fig. 6a, the tensile r-values are relatively high and remain approximately constant over the deformation range, consistent with previous observations reported by Kim et al.^7^. In contrast, the *r*-values from the equi-biaxial bulge test (Fig. 6b) are lower and exhibit a distinct evolution with increasing plastic strain, indicating a strain-path dependent anisotropic behavior.

To describe the hardening behavior, a Hockett-Sherby approximation^16^ was calibrated using the strain-stress data from the uniaxial tensile test in rolling direction, the fitted parameters are listed in Table 2.

**Table 2:** Hardening parameters for the utilized Hockett-Sherby approximation.

$\sigma _{sat}$ [MPa]	$\sigma _{yld}$ [MPa]	*m* [-]	*n* [-]
829.77	241.47	1.0704	0.4914

#### Yield locus calibration

The presented material values are then used to perform the calibration procedure at different levels of plastic strain. The parameter values corresponding to $a = 12$ are provided in Table 3. The parameters for the remaining exponent values can be found in the provided supplementary.

**Fig. 7:**
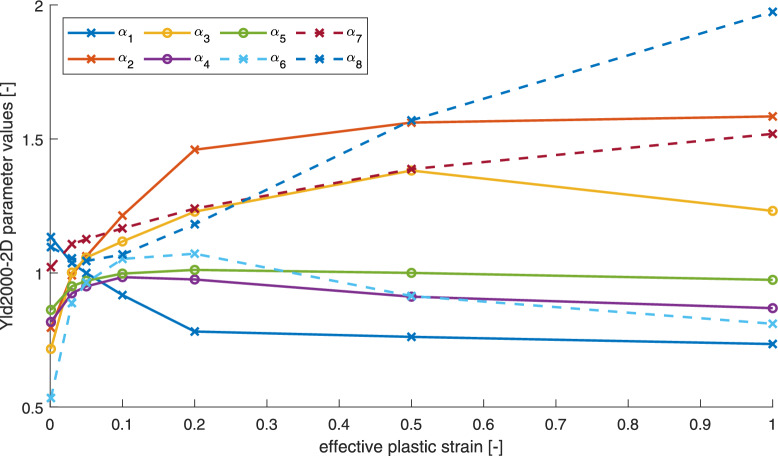
Dependence of the Yld2000-2D parameters on effective plastic strain for the exponent of $a=12$.

**Fig. 8:**
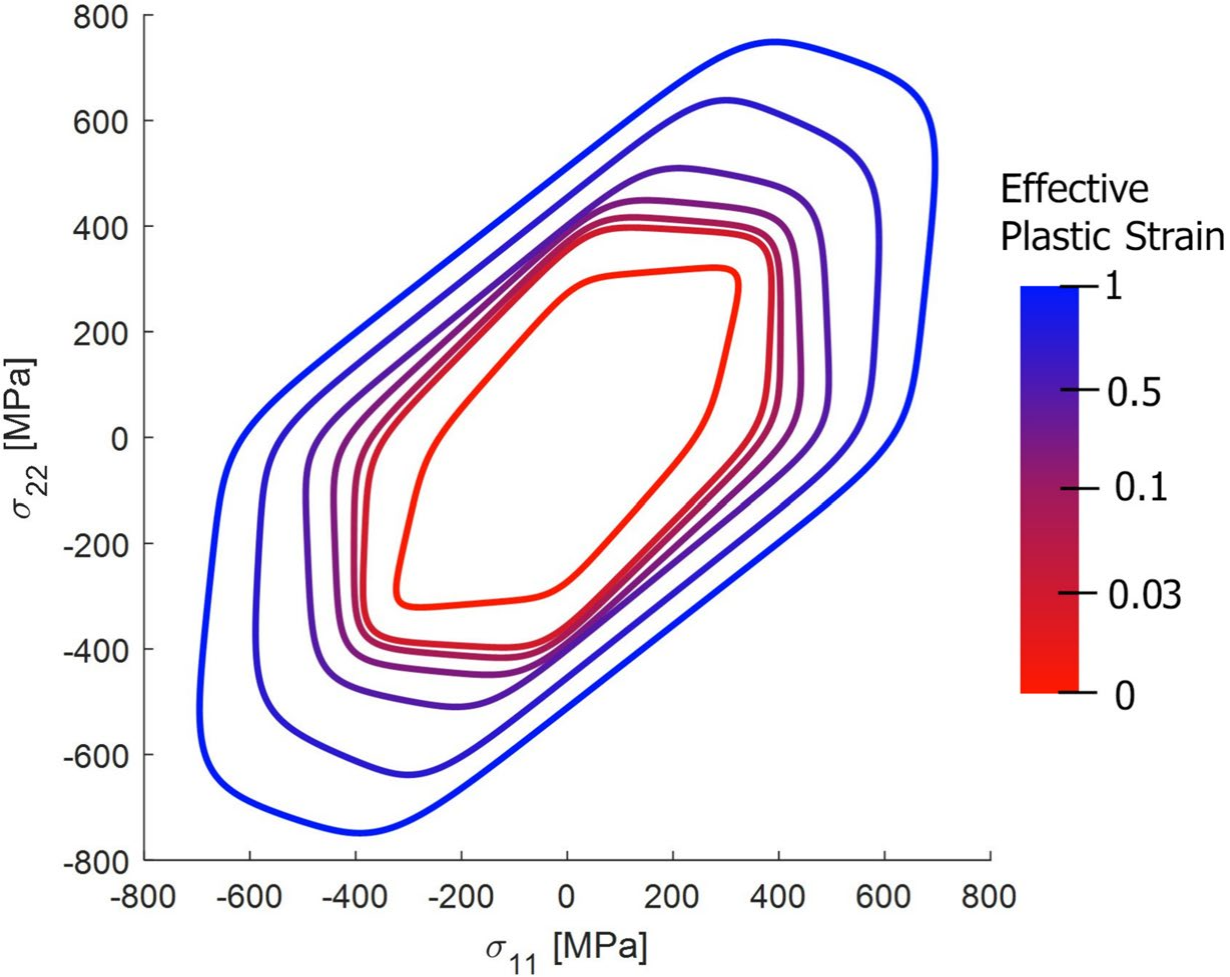
Evolution of the shape of the yld2000-2D surface as a result of distortional hardening for the exponent of $a=12$.

The evolution of the identified $\alpha _i$ parameters with increasing plastic strain is visualized in Fig. 7, illustrating the sensitivity of the yield locus shape to plastic deformation. Almost all parameter values seem to converge with increasing strain. This is in accordance with other results from literature. The only exception being the value for $\alpha _8$, which is still increasing at the plotted strain range. The exact reason for this is unknown. The impact of the parameter evolution on the shape of the yield surface can be seen in Fig. 8.

**Table 3:** Calibrated yield locus parameters for different plastic strains ranging from 0 to 1.

$\varepsilon _{pl} [-]$	$\varvec{\alpha }_1$	$\varvec{\alpha }_2$	$\varvec{\alpha }_3$	$\varvec{\alpha }_4$	$\varvec{\alpha }_5$	$\varvec{\alpha }_6$	$\varvec{\alpha }_7$	$\varvec{\alpha }_8$
0	1.1340	0.7961	0.7168	0.8176	0.8624	0.5337	1.0217	1.0967
0.03	1.0371	0.9914	1.0018	0.9243	0.9503	0.8879	1.1076	1.0546
0.05	0.9998	1.0623	1.0575	0.9501	0.9711	0.9646	1.1262	1.0449
0.1	0.9177	1.2143	1.1176	0.9845	0.9979	1.0526	1.1667	1.0690
0.2	0.7817	1.4599	1.2288	0.9759	1.0116	1.0720	1.2402	1.1820
0.5	0.7618	1.5611	1.3822	0.9117	1.0004	0.9141	1.3876	1.5692
1	0.7350	1.5841	1.2317	0.8689	0.9744	0.8106	1.5191	1.9741

**Fig. 9:**
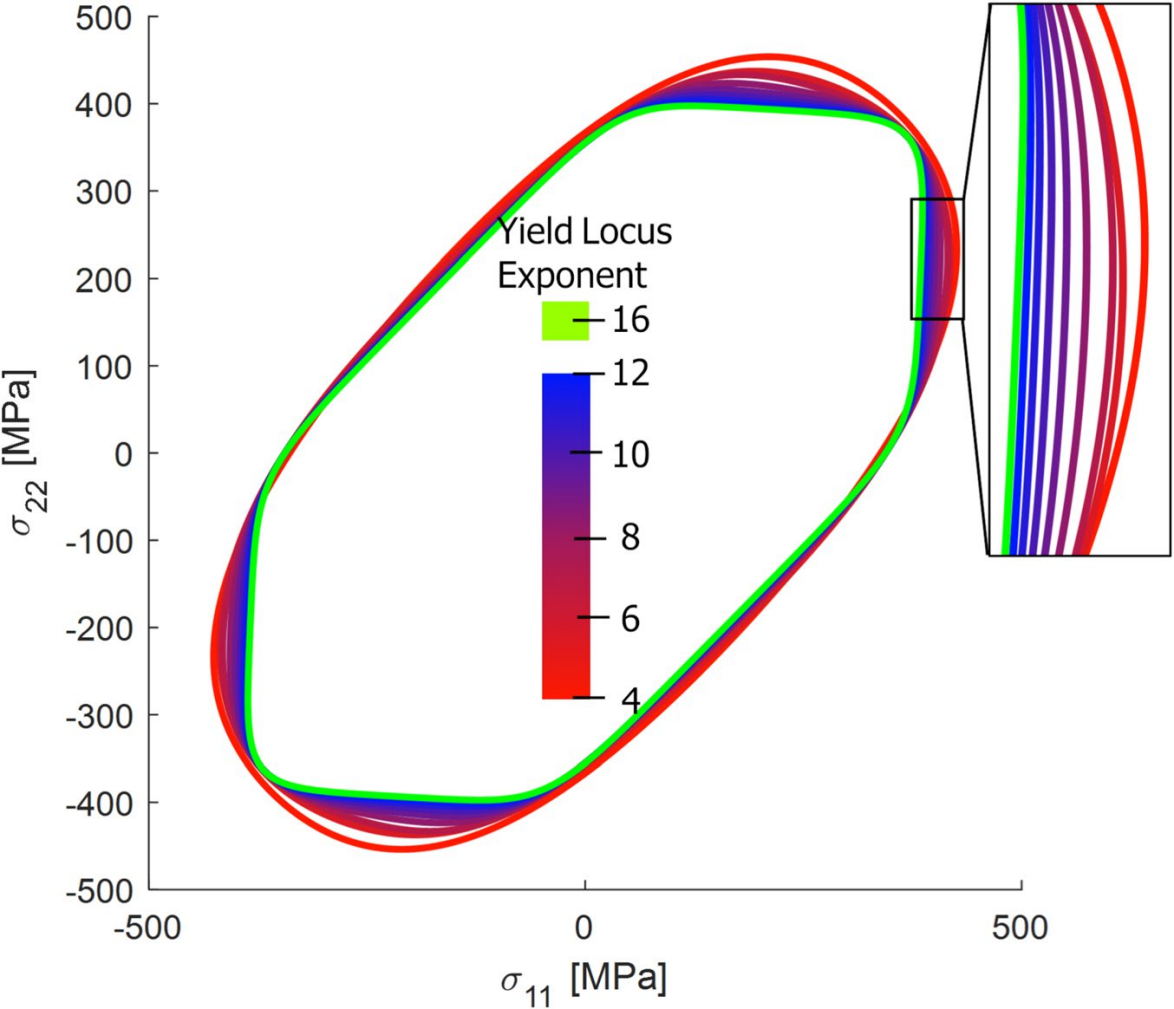
Influence of the yield locus exponent (*a*) on the shape of the Yld2000-2D yield function. The yield locus were plotted for parameters obtained at a plastic strain of 0.05.

Figure 9 highlights the influence of the yield function exponent *a* on the shape of the Yld2000-2D yield locus. As expected, within the calibration stress states, the yield loci are in close agreement. However, discrepancies arise in off-calibration regimes, particularly in plane-strain and tension-compression stress states. Increasing the exponent *a* results in a more faceted yield surface, trending towards the Tresca criterion, which is especially evident in the blue-shaded regions of the plot.

**Fig. 10:**
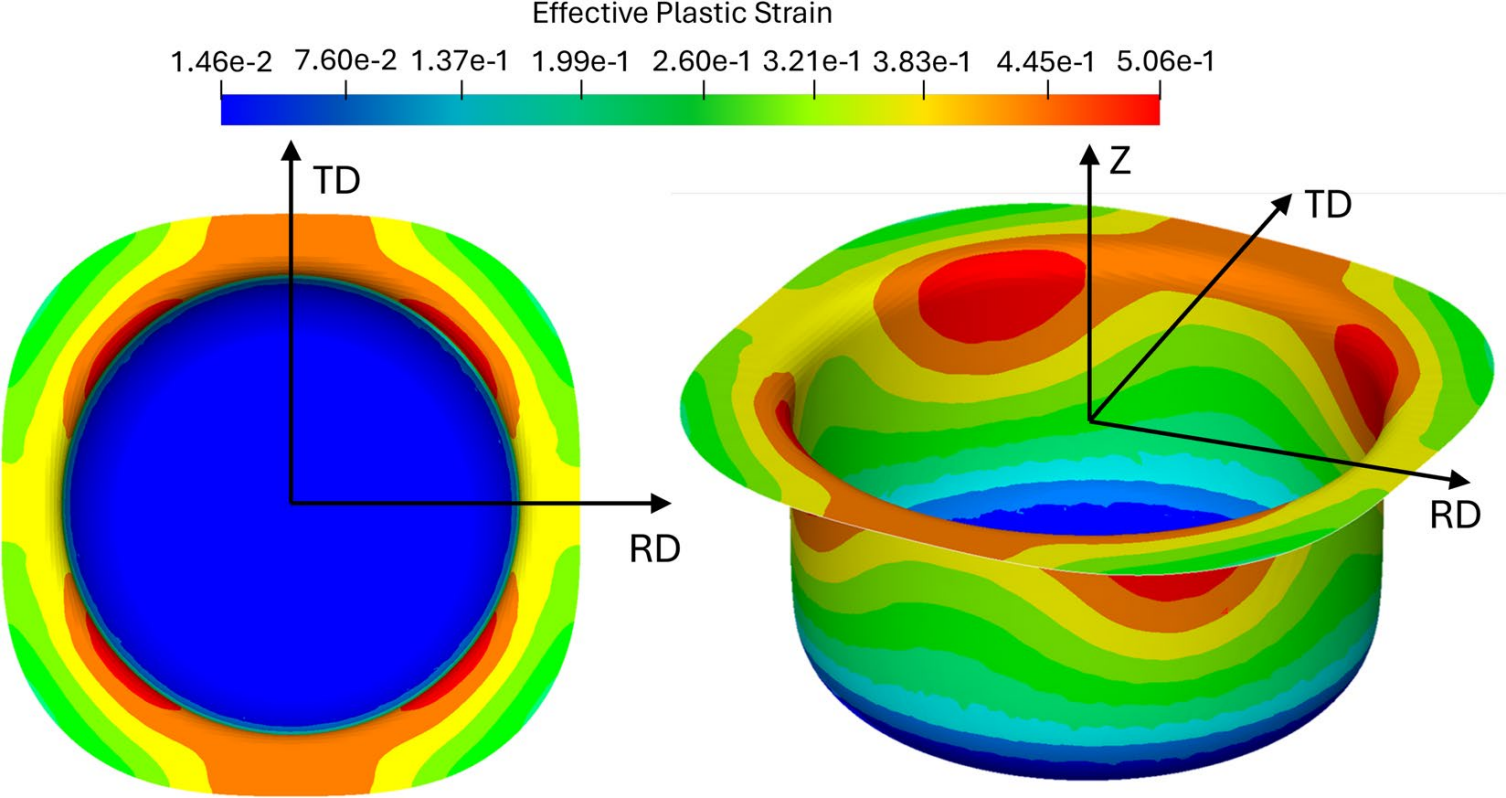
Effective plastic strain resulting from a simulation using the Yld2000-2D model with exponent a = 12, with the rolling direction (RD) and transversal direction (TD) indicated.

### Simulation results

Figure 10 depicts the effective plastic strain distribution from the deep drawing simulation employing a Yld2000-2D model with an exponent of $a=8$. The anisotropic response of the material is distinctly visible: the top-down view reveals non-uniform strain contours, with directional variation between RD (rolling direction) and TD (transverse direction). Strain localization occurs at angular positions influenced by the directional anisotropy of the titanium sheet. Notably, regions aligned at $45^\circ$ to RD show reduced draw-in, reflecting the underlying texture-induced anisotropy.

The 3D perspective (the right image in 10) further elucidates localized strain accumulation near the flange radius. This region undergoes complex deformation involving draw-in, tension-compression transitions, and bending, highlighting the necessity of a well-calibrated anisotropic yield model.

**Fig. 11:**
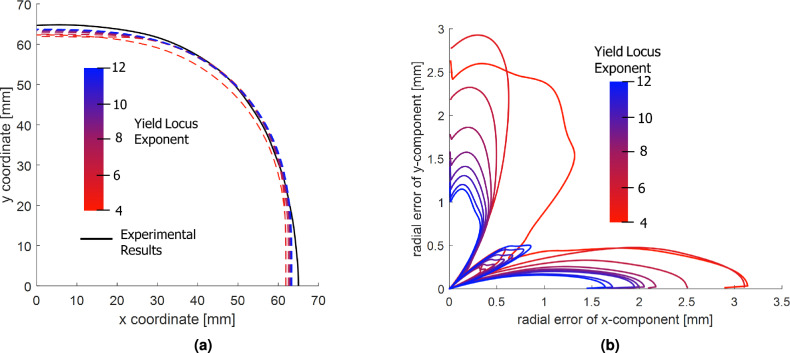
Accuracy of deformation prediction: **(a)** Comparison of experimental and predicted draw-in profiles. **(b)** Radial error distribution between RD and TD.

The predictive accuracy of the simulations is evaluated in Fig. 11a, where the simulated and experimental cup outlines are compared. All simulations (irrespective of *a* values) predict a slightly larger draw-in compared to the experiment, with the most significant differences appearing along RD. The model with lower exponent values tends to overestimate the draw-in, particularly at $0^\circ$ and $90^\circ$, whereas predictions at approximately $45^\circ$ align more closely with the experimental data. This behavior implies the sensitivity of the yield surface curvature to the exponent value and its impact on predicting material flow in anisotropic forming processes.

As shown in Fig. 11b, larger values of the yield locus exponent *a* lead to noticeably more accurate FE predictions of the cup radius, as evidenced by the reduction in radial error. Although the convergence trend is nonlinear, it remains consistent across all nine evaluated cases. Incremental improvements diminish as *a* increases from 4 to 12, indicating a saturation effect. The angular distribution of radial errors also reveals systematic patterns: the minimum and maximum errors consistently occur at approximately $0^\circ$ and $85^\circ$ relative to the rolling direction, respectively. These locations appear particularly sensitive to the degree of anisotropy captured by the chosen value of a. Specifically, the simulation at $a=4$ (worst case) exhibits a radial prediction that is approximately 6.5 times less accurate than that at $a=12$ (best case).

**Fig. 12:**
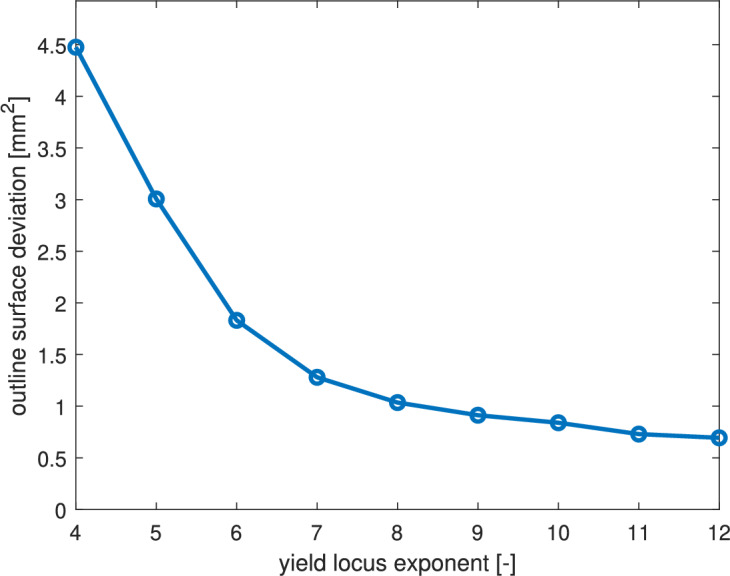
Radial MSE for each tested yield locus exponent *a*.

Fig. 12 summarizes the radial MSE as a function of *a*. A clear downward trend is observed, demonstrating that increasing the exponent leads to improved prediction accuracy in material flow. The most substantial reduction in error occurs between the lower values of *a*, particularly from 4 to 8, where the total draw-in deviation drops from approximately 4.5 mm^2^ to under 1 mm^2^. This indicates that smaller exponents severely underrepresent the anisotropic flow behavior of the titanium sheet, resulting in overpredicted draw-in.

As *a* increases beyond 8, improvements persist but at a slower rate. For $a>10$, the MSE curve begins to plateau, suggesting convergence of the numerical model toward the experimentally observed flow profile. This diminishing return indicates that, although higher exponents more accurately capture the stress state transitions (especially in plane-strain and biaxial regimes) there exists a practical upper bound beyond which further refinement has negligible benefit.

The results suggest that a yield locus which more closely resembles the Tresca model, creates more acccurate simulation results for metals consisting of a HCP crystal structure. A potential explanation for this could be due to the limited number of slip systems present in HCP crystals which cause the material to be more brittle than FCC or BCC structures resulting in a preference for the Tresca model. Further investigation and analysis is necessary to provide a funded reason for this trend.

From the test cases examined, it is therefore recommended to use a yield locus exponent $a\ge 9$ when applying the Yld2000-2D model to commercially pure titanium sheets under forming conditions. This range ensures a balance between model fidelity and computational efficiency, offering reliable prediction of anisotropic material behavior without unnecessary parameter inflation.

Furthermore, assuming the exponent value is strictly linked to the underlying microstructure as literature suggests, it can be assumed the presented findings are applicable to all metals with a HCP crystal structure, such as magnesium. This is further supported by the work of Badr et al.^12^, who came to the same optimal exponent value for a Ti64 sheet material. Nevertheless, proper investigation in different HCP materials is necessary to support this claim.

## Conclusion

This study investigated the influence of the yield locus exponent on the prediction accuracy of the draw-in during cup forming for commercially pure titanium sheets, addressing the gap in modeling the anisotropic behavior of such materials under forming conditions. The findings highlight the necessity of calibrating the yield locus exponent (*a*) to improve the predictive accuracy of forming simulations for HCP materials like titanium. Key takeaways include:

- The investigated values demonstrated a significant improvement in aligning FE simulations with experimental draw-in profiles, achieving a radial mean squared error of approximately 3% (relative to the cup radius) for $a > 9$.
- The results showed that larger exponents (*a*) consistently led to reduced radial errors in cup radius predictions, with the maximum error observed approximately in rolling direction of the sheet, and about 6.5 times larger for the lowest exponent investigated here ($a = 4$) compared to the highest one ($a = 12$).

Despite these advancements, the study has certain limitations. The analysis focused on a specific forming process (cup-drawing) and employed a simplified friction model, which may not adequately capture the complex interactions present in industrial forming scenarios. Additionally, the investigation relied on only two sets of experimental data, limiting its applicability to other HCP materials or varying processing conditions. Another limitation of this work is that the pronounced yielding asymmetry, stemming from the HCP crystal structure, is not taken into account. However, since the effect of the exponent on the yield locus shape of models that do include loading asymmetry, such as^10^, is the same as for the herein investigated yld2000-2D model, it is not expected to substantially change the findings of the current work. Nevertheless, a proper investigation confirming this assumption is required.

Future work should address these limitations by extending the findings to diverse forming processes, such as deep drawing or stretch forming, and incorporating more sophisticated friction models to better reflect real-world conditions. Expanding the investigation to include a broader range of HCP materials and anisotropic metals could establish a comprehensive database of yield locus exponents, which, in turn, enables more robust and versatile predictive models for manufacturing applications.

## Supplementary Information

Supplementary Information.

## Data Availability

All relevant data can be found in either the manuscript or the provided supplementary information files.
