# Supplementary material for: Effect of yield locus exponent on draw-in prediction during deep drawing of commercially pure titanium
Source: Sci Rep. 2025 Nov 11;15:39500. doi: 10.1038/s41598-025-23156-y (PMC12606351; doi:10.1038/s41598-025-23156-y)
Supplement: Supplementary file 1 — Supplementary Information. [file 41598_2025_23156_MOESM1_ESM.pdf]

Supplementary Information for: Determination of Optimal Yield Locus Exponent for Commercially Pure Titanium Sheets under Forming

Lukas Gassler[1,2,*], **Andreas Hirsch**[1], **and Mohamadreza Afrasiabi**[1]

[1]ETH Zurich, Advanced Manufacturing Lab, 8005 Zurich, Switzerland
[2]inspire AG, Computational Manufacturing Group, 8005 Zurich, Switzerland
[*]gasslerl@ethz.ch

ABSTRACT

Full Material Data

a=5

$\varepsilon_{pl}[-]$	α_1	α_2	α_3	α_4	α_5	α_6	α_7	α_8
0	1.0279	0.9399	0.9362	0.7912	0.8305	0.3176	1.0772	0.9441
0.03	0.9064	1.1573	1.4200	0.8616	0.8668	0.3609	1.1718	0.8855
0.05	0.9220	1.1777	1.4858	0.8831	0.8792	0.3982	1.1924	0.8708
0.1	0.9390	1.2127	1.5438	0.9072	0.8989	0.4302	1.2323	0.9010
0.2	0.9409	1.2582	1.5589	0.9185	0.9188	0.4524	1.3012	1.0307
0.5	0.9225	1.2749	1.4604	0.9042	0.9217	0.3918	1.4661	1.4612
1	0.8813	1.3315	1.4228	0.8883	0.9283	0.4261	1.6219	1.8862

Table 1. Calibrated yield locus parameters for different plastic strains ranging from 0 to 1 for exponent of 5.

a=6

$\varepsilon_{pl}[-]$	α_1	α_2	α_3	α_4	α_5	α_6	α_7	α_8
0	1.1204	0.8543	0.8248	0.8045	0.8380	0.4650	1.0608	0.9796
0.03	0.9977	1.0909	1.2056	0.8890	0.9152	0.7617	1.1532	0.9084
0.05	0.9415	1.1811	1.3190	0.9039	0.9282	0.7850	1.1729	0.8943
0.1	0.8329	1.3390	1.5558	0.9061	0.9147	0.5983	1.2130	0.9379
0.2	0.8462	1.3957	1.5726	0.9217	0.9428	0.6171	1.2824	1.0656
0.5	0.8483	1.4228	1.5284	0.9265	0.9509	0.5762	1.4457	1.4389
1	0.8031	1.4683	1.3826	0.8757	0.9454	0.5423	1.5968	1.9256

Table 2. Calibrated yield locus parameters for different plastic strains ranging from 0 to 1 for exponent of 6.

a=7

$\varepsilon_{pl}[-]$	α_1	α_2	α_3	α_4	α_5	α_6	α_7	α_8
0	1.1341	0.8295	0.7835	0.8091	0.8448	0.5008	1.0492	1.0125
0.03	1.0226	1.0512	1.1188	0.9003	0.9296	0.8380	1.1397	0.9486
0.05	0.9768	1.1327	1.2012	0.9192	0.9489	0.8997	1.1591	0.9337
0.1	0.8674	1.3117	1.3609	0.9314	0.9653	0.9125	1.2008	0.9532
0.2	0.7984	1.4591	1.5649	0.9177	0.9565	0.7317	1.2694	1.0994
0.5	0.8045	1.5005	1.5153	0.9227	0.9675	0.6845	1.4290	1.4667
1	0.7693	1.5305	1.3454	0.8651	0.9533	0.6157	1.5775	1.9710

Table 3. Calibrated yield locus parameters for different plastic strains ranging from 0 to 1 for exponent of 7.

a=8

$\varepsilon_{pl}[-]$	α_1	α_2	α_3	α_4	α_5	α_6	α_7	α_8
0	1.137 0	0.8168	0.7598	0.8119	0.8501	0.5159	1.0407	1.0379
0.03	1.0308	1.0297	1.0744	0.9078	0.9370	0.8644	1.1298	0.9804
0.05	0.9883	1.1074	1.1449	0.9289	0.9576	0.9351	1.1489	0.9670
0.1	0.8919	1.2753	1.2561	0.9496	0.9818	0.9957	1.1904	0.9855
0.2	0.7743	1.4854	1.5388	0.9105	0.9656	0.8119	1.2609	1.1238
0.5	0.7842	1.5354	1.4861	0.9161	0.9780	0.7584	1.4155	1.4971
1	0.7534	1.5587	1.3107	0.8585	0.9581	0.6684	1.5627	2.0018

Table 4. Calibrated yield locus parameters for different plastic strains ranging from 0 to 1 for exponent of 8.

a=9

$\varepsilon_{pl}[-]$	α_1	α_2	α_3	α_4	α_5	α_6	α_7	α_8
0	1.1371	0.8089	0.7439	0.8139	0.8542	0.5239	1.0342	1.0576
0.03	1.0343	1.0155	1.0465	0.9134	0.9419	0.8764	1.1222	1.0053
0.05	0.9938	1.0908	1.1107	0.9361	0.9628	0.9504	1.1412	0.9931
0.1	0.9032	1.2526	1.1993	0.9619	0.9890	1.0262	1.1823	1.0132
0.2	0.7612	1.4953	1.5109	0.9052	0.9725	0.8709	1.2544	1.1453
0.5	0.7740	1.5510	1.4576	0.9121	0.9859	0.8137	1.4068	1.5193
1	0.7443	1.5727	1.2781	0.8557	0.9615	0.7083	1.5516	2.0205

Table 5. Calibrated yield locus parameters for different plastic strains ranging from 0 to 1 for exponent of 9.

a=10

$\varepsilon_{pl}[-]$	α_1	α_2	α_3	α_4	α_5	α_6	α_7	α_8
0	1.1363	0.8034	0.7324	0.8154	0.8575	0.5286	1.0292	1.0733
0.03	1.0360	1.0053	1.0271	0.9178	0.9455	0.8826	1.1163	1.0252
0.05	0.9968	1.0788	1.0874	0.9418	0.9664	0.9581	1.1351	1.0140
0.1	0.9100	1.2364	1.1627	0.9712	0.9932	1.0406	1.1760	1.0356
0.2	0.7532	1.4981	1.4865	0.9023	0.9777	0.9153	1.2493	1.1635
0.5	0.7682	1.5578	1.4307	0.9103	0.9919	0.8558	1.3994	1.5387
1	0.7443	1.5767	1.2838	0.8645	0.9705	0.7660	1.5202	1.9534

Table 6. Calibrated yield locus parameters for different plastic strains ranging from 0 to 1 for exponent of 10.

a=11

$\varepsilon_{pl}[-]$	α_1	α_2	α_3	α_4	α_5	α_6	α_7	α_8
0	1.1352	0.7993	0.7237	0.8166	0.8602	0.5316	1.0251	1.0861
0.03	1.0368	0.9975	1.0128	0.9214	0.9482	0.8860	1.1115	1.0413
0.05	0.9987	1.0696	1.0705	0.9463	0.9690	0.9623	1.1302	1.0309
0.1	0.9145	1.2240	1.1369	0.9785	0.9960	1.0483	1.1709	1.0539
0.2	0.7733	1.4747	1.2975	0.9551	1.0056	1.0453	1.2446	1.1669
0.5	0.7645	1.5605	1.4054	0.9103	0.9966	0.8882	1.3907	1.5592
1	0.7325	1.5861	1.2211	0.8576	0.9660	0.7635	1.5496	2.0438

Table 7. Calibrated yield locus parameters for different plastic strains ranging from 0 to 1 for exponent of 11.

a=12

$\varepsilon_{pl}[-]$	α_1	α_2	α_3	α_4	α_5	α_6	α_7	α_8
0	1.1340	0.7961	0.7168	0.8176	0.8624	0.5337	1.0217	1.0967
0.03	1.0371	0.9914	1.0018	0.9243	0.9503	0.8879	1.1076	1.0546
0.05	0.9998	1.0623	1.0575	0.9501	0.9711	0.9646	1.1262	1.0449
0.1	0.9177	1.2143	1.1176	0.9845	0.9979	1.0526	1.1667	1.0690
0.2	0.7817	1.4599	1.2288	0.9759	1.0116	1.0720	1.2402	1.1820
0.5	0.7618	1.5611	1.3822	0.9117	1.0004	.91410	1.3876	1.5692
1	0.7350	1.5841	1.2317	0.8689	0.9744	0.8106	1.5191	1.9741

Table 8. Calibrated yield locus parameters for different plastic strains ranging from 0 to 1 for exponent of 12.
